# Periodontal regenerative therapy with enamel matrix derivative in the treatment of intrabony defects: a prospective 2-year study

**DOI:** 10.1186/s13104-017-2572-2

**Published:** 2017-07-06

**Authors:** Fumi Seshima, Hideto Aoki, Takahiro Takeuchi, Eiichi Suzuki, Daisuke Irokawa, Asako Makino-Oi, Hiroki Sugito, Sachiyo Tomita, Atsushi Saito

**Affiliations:** 1 Department of Periodontology, Tokyo Dental College, Tokyo, Japan; 2 Department of Operative Dentistry, Cariology and Pulp Biology, Tokyo Dental College, Tokyo, Japan; 3 Department of Dental Hygiene, Tokyo Dental Junior College, Tokyo, Japan; 4 Oral Health Science Center, Tokyo Dental College, Tokyo, Japan

**Keywords:** Periodontitis, Periodontal regeneration, Enamel matrix derivative

## Abstract

**Objective:**

To date, enamel matrix derivative (EMD) has been considered to be one of the few biomaterials for clinical use capable of demonstrating true periodontal regeneration. The aim of this two-center prospective clinical study was to evaluate 2-year outcome of periodontal regenerative therapy using EMD in the treatment of intrabony defects, performed as an ‘advanced medical treatment’ under the national healthcare system in Japan.

**Results:**

Patients with chronic periodontitis who have completed initial periodontal therapy at either of the two dental school clinics were enrolled. Each contributed at least one intrabony defect of ≥3 mm in depth. During surgery, EMD was applied to the defect following debridement. Twenty-two participants (mean age 55.2 years old, 9 men and 13 women) completed 2-year reevaluation, and a total of 42 defects were subjected to data analysis. Mean gains in clinical attachment level (CAL) at 1 and 2 years were 2.9 mm (38% of baseline CAL) and 3.1 mm (41%), respectively, both showing a significant improvement from baseline. There was also a significant reduction in probing depth (PD): mean reductions at 1 and 2 years were 3.2 and 3.3 mm, respectively. There was a progressive improvement in the mean percentages of bone fill from 26% at 1 year to 36% at 2 years. No significant difference in CAL gain at 2 years was found between 3-wall bone defects and other defect types combined. In multiple regression analysis, the baseline PD was significantly associated with CAL gain at 2 years. In this population of patients, the treatment of intrabony defects with EMD yielded clinically favorable outcomes, as assessed by periodontal and radiographical parameters, over a period of 2 years.

**Electronic supplementary material:**

The online version of this article (doi:10.1186/s13104-017-2572-2) contains supplementary material, which is available to authorized users.

## Introduction

Periodontitis is an inflammatory disease of tooth supporting tissues induced by dental plaque biofilm [1]. Cumulative evidence indicates that it is a dysbiotic disease which could induce a negative impact on systemic health [2]. In patients with moderate to advanced periodontitis, surgical periodontal therapy is often necessary, following non-surgical intervention.

Enamel matrix derivative (EMD) [3] has been used for the purpose of periodontal regeneration for over two decades with favorable results [4–8]. It is considered to be one of the few biomaterials available for clinical use capable of histologically demonstrating true periodontal regeneration [8].

In Japan, under the system of ‘advanced medical treatment’, the use of treatment that is not covered by national healthcare insurance is permitted together with covered treatment in special cases. The use of EMD has been approved as an ‘advanced medical treatment: bio-regeneration method’ by the Ministry of Health, Labor and Welfare. This increased the opportunity for periodontitis patients to seek the regenerative therapy. Tokyo Dental College Chiba Hospital and Suidobashi Hospital received a formal approval for its use as the advanced medical treatment (on April 2008 and January 2011, respectively). Prior to this, we conducted a retrospective study evaluating the clinical outcome of treatment of intrabony defects with EMD [9]. We felt that it is our responsibility to longitudinally evaluate the regenerative therapy with EMD performed as the advanced medical treatment.

In this two-center prospective clinical study, we aimed to evaluate 2-year clinical outcome following surgical treatment of intrabony periodontal defects with EMD, performed as an ‘advanced medical treatment’ under the national healthcare system in Japan.

## Methods

### Study design and participants

This prospective, two-center, clinical study is part of our ongoing research on the longitudinal outcome of regenerative therapy using EMD alone, performed as an ‘advanced medical treatment’. The participants were recruited from patients with chronic periodontitis [10], who visited Tokyo Dental College Suidobashi Hospital (Tokyo, Japan) or Tokyo Dental College Chiba Hospital (Chiba, Japan).

### Inclusion and exclusion criteria

Inclusion criteria consisted of having interproximal sites with probing depth (PD) ≥6 mm, at least one intrabony defect ≥3 mm in depth in interproximal area of teeth and adequate level of plaque control (mean Plaque Index ≤1) [11]. Participants must have received initial periodontal therapy.

Exclusion criteria were the presence of uncontrolled systemic diseases, smokers, allergy to common medications, concurrent or previous anti-resorptive agents such as bisphosphonate or other drug therapy, current pregnancy or lactation and contraindications for dental and/or surgical interventions. Patients under 20 years old or with furcation involvements at target sites are excluded.

### Clinical examination

The following parameters were recorded by trained, calibrated examiners at baseline essentially as described previously [12]: PD, gingival recession (GR), clinical attachment level (CAL), bleeding on probing (BOP), and tooth mobility (TM). Reevaluations were performed at 1 and 2 years after surgery.

### Radiographic assessment

Semi-standardized radiographs were taken using film holders with customized occlusal stents as described previously [12]. Measurements from the radiographs were made using the Schei Ruler Technique [13]. The degree of change in the tooth axis heights between the cemento-enamel junction (CEJ) and bottom of the bone defect was defined as linear alveolar bone growth, and the percentage of bone fill was calculated by dividing the linear bone growth by the bone defect depth at baseline.

### Surgical procedures

Following local infiltration anaesthesia, a full-thickness flap was used to gain access to the defect area. An effort was made to preserve papilla as much as possible during incision. Granulation tissue was removed, and scaling and root planning was performed. Following root conditioning and rinsing, 0.3 or 0.7 ml EMD (Emdogain^®^ Gel, BIORA AB/Straumann, Switzerland) was applied. No bone graft or other supplementary modalities were used. The flaps were then replaced and sutured with a PTFE non-resorbable sutures (Tefdesser II, 5-0, Kono Seisakusho, Chiba, Japan). No periodontal dressing was used. Eight periodontists with at least 3 years of periodontal training performed surgery.

The intrabony component of the defect (INTRA) was calculated as described previously [12]. The number of bone walls was also registered.

### Postsurgical care and maintenance

The patients received antimicrobial agents (typically cefdinir 300 mg/day, for 4 days). Standard analgesic was prescribed as necessary. They rinsed twice daily with an antimicrobial mouthrinse and started gentle wiping of the operated area with a soft toothbrush from the 3rd day.

The sutures were removed after 10–14 days. Professional supragingival tooth cleaning was performed at weeks 1, 2 and 4. Subsequently, all patients were placed on the maintenance program.

### Data management and statistical analysis

Each patient contributed one to multiple defects. The primary endpoint variable was the change in CAL. Friedman test with Dunn’s post hoc test was used to assess changes in data over time. Comparisons for BOP data were made by Fisher’s exact test. Difference in CAL gain between two different defect types was sought by Mann–Whitney U test. Correlation between variables was analyzed by Spearman’s rank correlation. Multiple regression analysis was performed to determine contributions of the explanatory baseline variables. A software package (InStat version 3.10 for Windows, GraphPad Software, La Jolla, CA, USA) was used. Statistical significance was defined as a *p* value of less than 0.05.

## Results

### Participants and baseline clinical parameters

Twenty-two patients (mean age 55.2 years old, 9 men and 13 women) completed 2-year reevaluation. Forty-two defects were subjected to data analysis. Treated teeth comprised 9 incisors or canines (6 maxillary, 3 mandibular), 9 premolars (4 maxillary, 5 mandibular) and 24 molars (11 maxillary, 13 mandibular).

No adverse events were observed in the participants throughout the study.

### Intrasurgical parameters

The mean value for INTRA was 5.0 ± 1.4 mm. The defect type comprised the following: 1 and 3-wall combination: 2, 2-wall: 8, 2 and 3-wall combination: 8, 3-wall: 26.

### Change in clinical parameters

At 1 year postoperatively, a significant improvement in CAL from baseline was observed (*p* < 0.01) (Table 1). An improvement from baseline was also observed at 2 years (*p* < 0.01). The mean gains in CAL (primary endpoint) at 1 and 2 years were 2.9 ± 1.2 mm (range 0.0–6.0 mm) and 3.1 ± 1.3 mm (range 0.0–7.0 mm), respectively. No significant difference in CAL gain was observed between at 1 and 2 years. Percentages of CAL gains at 1 and 2 years, relative to baseline CAL, were 38.1 and 40.7%, respectively.

**Table 1:** Change in clinical parameters

Variable	Baseline	1-year	2-year
CAL (mm)^a^	7.6 ± 1.8 (7.1–8.1)	4.8 ± 1.3^**^ (4.2–5.3)	4.5 ± 1.5^**^ (4.1–5.1)
PD (mm)^a^	6.8 ± 1.2 (6.4–7.1)	3.3 ± 1.0^**^ (3.1–3.6)	3.2 ± 1.0^**^ (3.0–3.4)
BOP (mean %)	42.9	2.3^#^	9.5^#^
TM^b^	0.2 ± 0.5	0.1 ± 0.3	0.1 ± 0.3

*CAL* clinical attachment level, *PD* probing depth, *BOP* bleeding on probing, *TM* tooth mobility

^**^ *p* < 0.01, significantly different from baseline, by Friedman test with Dunn’s multiple comparisons test

^#^ *p* < 0.05, significantly different from baseline, by Fisher’s exact test

^a^mean ± SD (lower 95% confidence interval—upper 95% confidence interval), ^b^mean ± SD

**Fig. 1:**
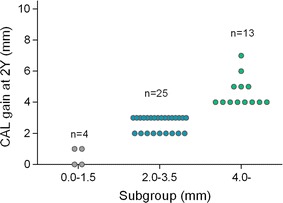
Distribution of CAL gain values at the treated sites 2 years postoperatively (n = 42 sites). *CAL* clinical attachment level

Distribution of CAL gain values at 2 years is shown in Fig. 1. CAL gain was noted at 41 sites and no change was found at one site.

Among the secondary endpoints, a significant improvement in PD from baseline was noted at 1 and 2 years (Table 1). No significant difference in PD was observed between at 1 and 2 years. Percentages of PD reductions at 1 and 2 years were 47.1 and 48.5%, respectively. As for BOP, a significant difference from baseline was noted at 1 and 2 years. No significant difference from baseline in TM score was noted at 1 and 2 years.

**Fig. 2:**
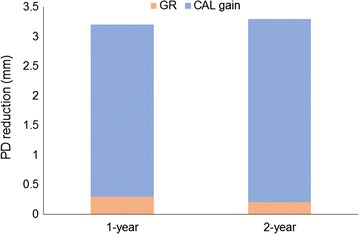
Contribution of GR and CAL gain to PD reduction at 2 years. Mean values are shown. *GR* gingival recession, *CAL* clinical attachment level

At both 1 and 2 years, the contribution of GR to the PD reduction was minimal (Fig. 2).

### Assessment of bone fill

At baseline, mean value of the percentage of bone loss was 47.5 ± 13.3%. The bone fill at 1 and 2 years postoperatively were 25.6 ± 12.9 and 36.2 ± 14.6%, respectively.

### Configuration of treated defects and CAL gain at 2 years

**Fig. 3:**
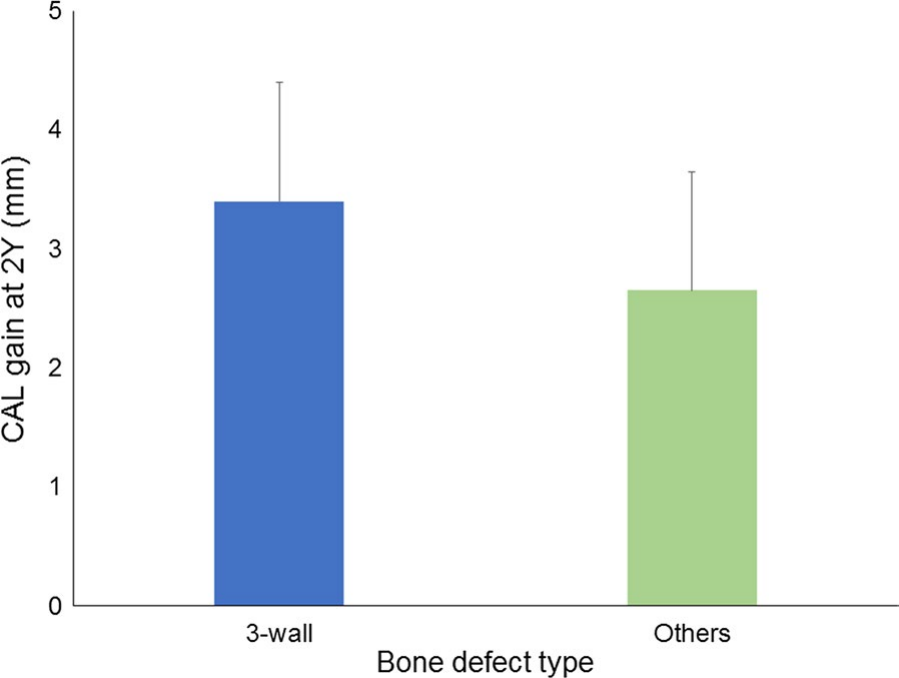
Configuration of treated defects and CAL gain at 2 years. Data shown as mean ± SD. *CAL* clinical attachment level

When the values for CAL gain at 2 years were compared between 3-wall defects (contained defects) and others (1-wall, 2-wall, and combination defects), no significant difference was observed between groups (*p* = 0.13, by Mann–Whitney U test) (Fig. 3).

### Relationship between CAL gain at 2 years and baseline variables

A significant positive correlation was found with baseline PD and patient age (Additional file 1: Table S1). In multiple regression analysis, the baseline PD was significantly associated with CAL gain at 2 years (Table 2).

**Table 2:** Multiple regression analysis for the association with CAL gain at 2 years

Baseline variable	Coefficient	Confidence interval	*t* ratio	*p*
CAL (mm)	−0.032	−0.344–0.281	0.206	0.838
PD (mm)	0.508	0.004–1.011	*2.045*	*0.048*
TM	–0.385	−1.123–0.354	1.058	0.297
INTRA (mm)	0.260	−0.073–0.592	1.584	0.122
Patient age	0.020	−0.014–0.053	1.16	0.242

Dependent variable: CAL gain at 2 years; R^2^ = 0.493. Significant association is indicated in italics

*CAL* clinical attachment level, *PD* probing depth, *TM* tooth mobility, *INTRA* intrabony component

### Treatment case

A representative case is shown in Additional file 2: Figure S1.

## Discussion

In this study, we evaluated clinically the 2-year outcome of periodontal regenerative therapy using EMD alone, performed as an ‘advanced medical treatment’, in the treatment of intrabony defects. The EMD therapy yielded statistically significant gains in CAL and reductions in PD at 2 years, when compared with the preoperative data.

The mean value of the primary endpoint, CAL gain, was 2.9 mm at 1 year. This value is slightly smaller than the value of 3.2 mm reported in a meta-analysis on the management of 317 angular bone defects with EMD during an observation period ranging from 6 months to 1 year [14] and the value of 3.1 mm reported in a multi-center study [15]. At 2 years, the mean value was 3.1 mm. This was comparable to that (3.2 mm) reported in our previous smaller-scale retrospective study [9] and other studies [15, 16].

A systematic review of the treatment of intrabony defects with EMD reported a significant additional gain in CAL of 1.3 mm compared with open-flap debridement, but no significant difference compared with resorbable membranes was shown [17]. In our previous study of the periodontal regenerative study using a deproteinized bovine bone mineral in combination with a collagen membrane [12], mean gain in CAL at 2.5 years was 1.4 mm, which is smaller than the 2-year value in the present study. Although it is difficult to directly compare these data, it is remarkable that the EMD therapy yielded such result without the use of any bone substitutes.

In this study, the mean preoperative (baseline) PD value was 6.8 mm. In the multiple regression analysis, baseline PD value was significantly associated with the CAL gain at 2 years. Similarly, other studies [6, 18] showed that deeper pockets gave significantly more CAL gain. These suggest that caution should be exercised when comparing the values of CAL gain in different studies.

Kitamura et al. [19] reported that the percentage of bone fill at 36 weeks following EMD therapy was 23%, which was comparable to our 1-year value of 26%. There was a progressive improvement in bone fill from at 1 year to 36% at 2 years. It has been reported that distinct radiographical bone fill was observed at as early as 5–6 months after the EMD therapy [20, 21]. Heijl et al. [20] also reported that further bone gain may be expected for as long as 3 years, which collaborates our findings.

Our multiple regression analysis showed that there was no significant association between INTRA and the CAL gain at 2 years. When the CAL gain values were compared between 3-wall defect (contained defect) and other defect types combined (uncontained defects), no significant difference was observed. Configuration of osseous defect has been shown to be an important determinant in EMD therapy [22]. Reflecting this, in a recent study of the use of EMD in the treatment of non-contained (1- and 2-wall) infrabony defects [23], the mean CAL gain at 1 year was shown to be 2.7 mm. This CAL gain value is relatively modest, considering that the mean PD at baseline was 7.9 mm, which was much greater than the value in this study. In our analysis, defect types other than 3-wall were combined due to the small sample size. It is necessary to evaluate the influence of bone defect configuration on longitudinal outcome of the use of EMD alone.

## Limitations

In this study, sample size was relatively small. The study design was single-arm with no control group for direct comparison. The surgeries were performed by eight periodontists with various clinical experience levels.

## Additional files

**Additional file 1: Table S1.** Correlations between baseline valuables and CAL gain at 2 years. *r*, Spearman coefficient. Significant differences are indicated in **bold**. CAL, clinical attachment level; PD, probing depth; TM, tooth mobility; INTRA, intrabony component.

**Additional file 2: Figure S1.** A representative treatment case. 53-year-old woman with severe chronic periodontitis. **a.** Preoperative clinical view. PD 7.0 mm, CAL 8.0 mm, INTRA 6.0 mm. b. During surgery; INTRA of the defect was 6.0 mm. **c.** Preoperative (baseline) radiograph, in the distal aspect of the mandibular right second molar. Angular bony defect is evident. **d.** Radiograph after 1 year. An improvement in radiolucency can be observed in the distal aspect. **e.** Radiograph after 2 year, showing further improvement in the distal aspect. PD 4.0 mm, CAL 4.0 mm.

## Abbreviations

EMD: enamel matrix derivative
CAL: clinical attachment level
PD: probing depth
BOP: bleeding on probing
TM: tooth mobility
GR: gingival recession
INTRA: intrabony component
