# Supplementary material for: Periodontal regenerative therapy with enamel matrix derivative in the treatment of intrabony defects: a prospective 2-year study
Source: BMC Res Notes. 2017 Jul 6;10:256. doi: 10.1186/s13104-017-2572-2 (PMC5501118; doi:10.1186/s13104-017-2572-2)
Supplement: Supplementary file 1 — Additional file 1: Table S1. Correlations between baseline valuables and CAL gain at 2 years. r, Spearman coefficient. Significant differences are indicated in bold. CAL, clinical attachment level; PD, probing depth; TM, tooth mobility; INTRA, intrabony component. [file 13104_2017_2572_MOESM1_ESM.docx]

Table S1. Correlations between baseline valuables and CAL gain at 2 years

|  | CAL gain | |
| --- | --- | --- |
| Baseline variable | *r* | *P* |
| CAL | 0.273 | 0.081 |
| PD | **0.425** | **0.006** |
| TM | -0.062 | 0.677 |
| INTRA | 0.132 | 0.376 |
| Patient age | **0.287** | **0.048** |

*r*, Spearman coefficient. Significant differences are indicated in **bold**.

CAL, clinical attachment level; PD, probing depth; TM, tooth mobility; INTRA, intrabony component
