# Supplementary material for: Periodontal regenerative therapy with enamel matrix derivative in the treatment of intrabony defects: a prospective 2-year study
Source: BMC Res Notes. 2017 Jul 6;10:256. doi: 10.1186/s13104-017-2572-2 (PMC5501118; doi:10.1186/s13104-017-2572-2)
Supplement: Supplementary file 2 — Additional file 2: Figure S1. A representative treatment case. 53-year-old woman with severe chronic periodontitis. a. Preoperative clinical view. PD 7.0 mm, CAL 8.0 mm, INTRA 6.0 mm. b. During surgery; INTRA of the defect was 6.0 mm. c. Preoperative (baseline) radiograph, in the distal aspect of the mandibular right second molar. Angular bony defect is evident. d. Radiograph after 1 year. An improvement in radiolucency can be observed in the distal aspect. e. Radiograph after 2 year, showing further improvement in the distal aspect. PD 4.0 mm, CAL 4.0 mm. [file 13104_2017_2572_MOESM2_ESM.pdf]

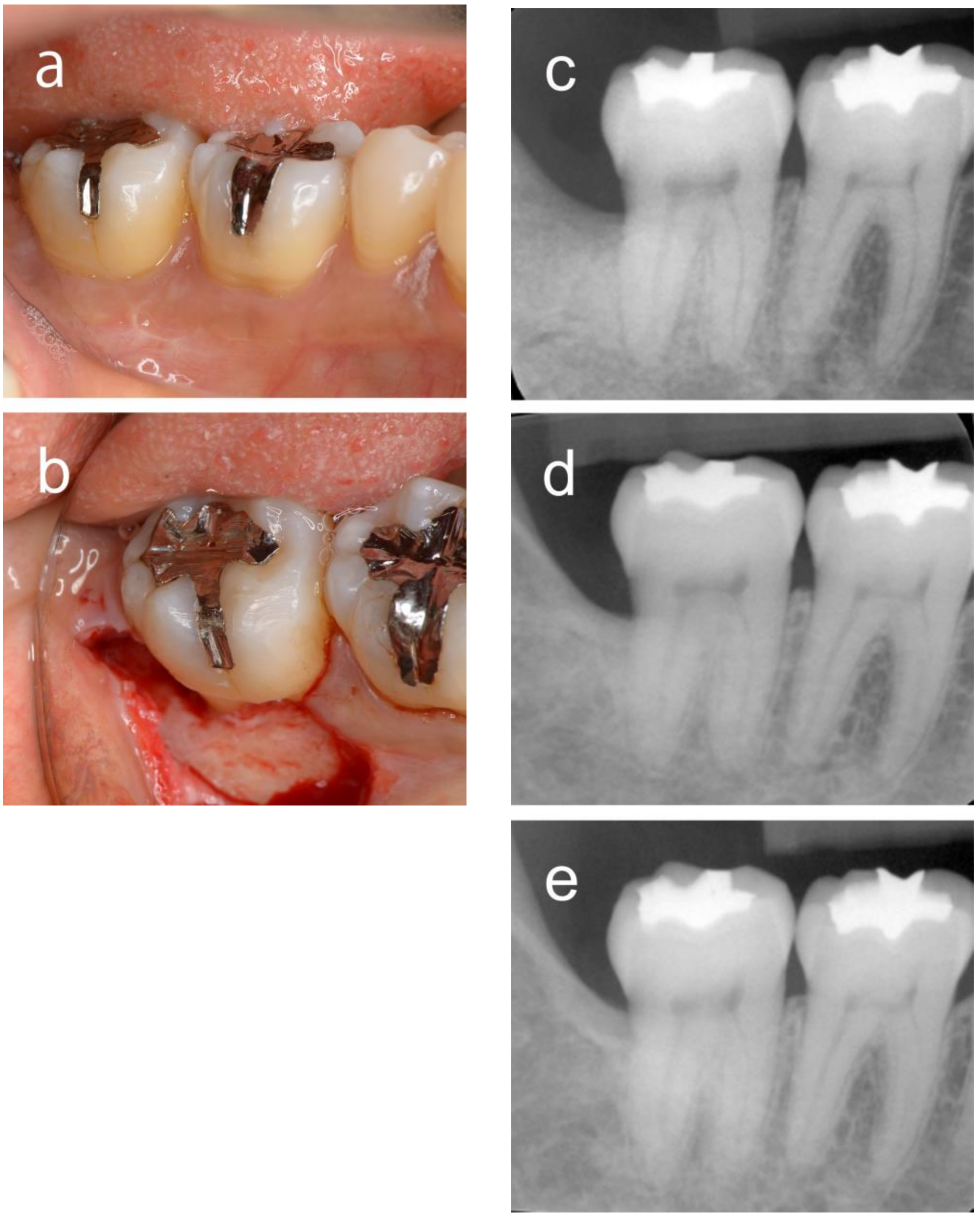

Fig. S1. A representative treatment case. 53-year-old woman with severe chronic periodontitis.

a. Preoperative clinical view. PD 7.0 mm, CAL 8.0 mm, INTRA 6.0 mm. b. During surgery; INTRA of the defect was 6.0 mm. **c.** Preoperative (baseline) radiograph, in the distal aspect of the mandibular right second molar. Vertical bone defect is evident. **d.** Radiograph after 1 year. An improvement in radiolucency can be observed in the distal aspect. **e.** Radiograph after 2 year, showing further improvement in the distal aspect. PD 4.0mm, CAL 4.0 mm.
